# Training LEADers to Accelerate Global Mental Health Disparities Research (LEAD) Program: A Research Training Program Protocol

**DOI:** 10.3389/fpubh.2021.749627

**Published:** 2021-11-10

**Authors:** Ozge Sensoy Bahar, Patricia Cavazos-Rehg, Fred M. Ssewamala, Betsy Abente, Laura Peer, Proscovia Nabunya, Lourdes E. Soto de Laurido, Theresa S. Betancourt, Arvin Bhana, Tonya Edmond

**Affiliations:** ^1^Brown School, Washington University in St. Louis, St. Louis, MO, United States; ^2^Department of Psychiatry, School of Medicine, Washington University in St. Louis, St. Louis, MO, United States; ^3^School of Health Professions, Medical Sciences Campus, University of Puerto Rico, San Juan, Puerto Rico; ^4^School of Social Work, Boston College, Boston, MA, United States; ^5^South African Medical Research Council, Cape Town, South Africa; ^6^Centre for Rural Health-School of Nursing & Public Health, University of KwaZulu-Natal, Durban, South Africa

**Keywords:** global mental health, training, disparities, research capacity building, across the life span

## Abstract

**Background:** There is a critical need to address mental health needs across the globe, especially in low and middle-income countries where mental health disparities are pervasive, including among children. The global mental health disparities suggest an imperative for culturally and contextually-congruent mental health services models that expand upon the existing services and interventions for these groups. Rigorous research is a key tool in providing the scientific evidence to inform public policy and practice efforts to effectively address these needs. Yet, there is a limited number of researchers, especially those from diverse backgrounds, who study these issues. In this paper, we describe the “*Training* ***LEAD****ers to Accelerate Global Mental Health Disparities Research”* (LEAD) program, a research training program funded by the National Institute on Minority Health and Health Disparities and focused on global mental health disparities research for early career researchers from under-represented minority groups.

**Methods:** The LEAD program is designed as a two-phase training program for advanced pre-doctoral students, postdoctoral fellows, and junior faculty from diverse backgrounds in the U.S., including groups underrepresented in biomedical, behavioral, clinical and social sciences research, interested in global mental health disparities research. Trainees are matched with mentors and participate in an intensive 12-week program.

**Discussion:** The LEAD program seeks to provide a robust platform for the development, implementation and expansion of evidence-based culturally and contextually-congruent interventions and services models addressing global mental health disparities across the life cycle, especially in low-resource communities in the global context. By producing a sustainable network of well-trained investigators from underrepresented backgrounds, LEAD will potentially contribute to the shared lessons and efforts relevant to addressing global mental health disparities and improving care for vulnerable populations in low-resource settings.

## Introduction

There is a critical need to address mental health needs across the globe, especially in low and middle-income countries where mental health disparities are pervasive, including among children. For instance, 90% of randomized clinical trials testing mental health interventions for children have been conducted in high-income countries (1), while 90% of the children live in low and middle-income countries (1, 2). Rigorous research is a key tool in providing the scientific evidence to inform policy and practice efforts to effectively address these needs. Yet, there is a limited number of researchers, especially those from diverse backgrounds who study these issues (3, 4). Hence, there is vital need for researchers from diverse backgrounds trained in studying mental health disparities in the global context, especially in low and middle-income countries.

There are several compelling reasons to increase the number of underrepresented researchers conducting mental health disparity research, including mental health in low-resource communities in the global context. First, the National Healthcare Disparities Report 2018 found that minority and low-income groups continue to struggle with healthcare quality and access (5). Healthcare disparities are particularly magnified within the field of mental health. Compared with the majority population, members of racial and ethnic minority (REM) groups, both in the U.S. and globally, are less likely to have access to mental health services, less likely to use community mental health services, more likely to use inpatient hospitalization and emergency rooms, and more likely to receive lower quality care (6). Second, globally, there are significant gaps in infrastructure and government investment in mental health. According to a report by the World Health Organization (WHO), the budgetary investment in mental health especially in low and middle-income countries remains significantly low and treatment gaps in mental health continue to remain high (7).

In addition, mental disorders are among the leading causes of disability in the world (8). Thus, improved strategies to reduce their burden are needed through appropriate intervention strategies and training programs. Finally, the 2014 WHO report on social determinants of global mental health points to the fact that some groups are at higher risk of mental disorders because of greater exposure and vulnerability to unfavorable social, economic, and environmental circumstances (9).

Within this context, child and adolescent mental health are particularly critical. Globally, 10–20% of children and adolescents experience mental health conditions (4). Children and adolescents in Sub-Saharan Africa (SSA), the poorest region in the world, comprise half of the total regional population; yet mental health services are severely under-equipped to meet their needs (3, 10). Even in a developed country like the U.S., efforts to scale up child mental health evidence-based practices have been impeded by serious challenges related to uptake, implementation, and integration as well as challenges due to limited human resources for mental health services needs of diverse groups (11, 12).

The continued global mental health disparities suggest an imperative for culturally and contextually-congruent mental health services models that expand upon the existing services and interventions typically used with these groups. Against that backdrop, trainees and mental health practitioners have called for shared lessons and efforts, arguing that lessons learned from global mental health efforts in low and middle-income countries dominated by the global south, including SSA, may be relevant to addressing global mental health disparities, and improving overall care for vulnerable populations in high-income countries, including the U.S. (13).

In this paper, we describe the training program in minority health and health disparities research, funded by the National Institute on Minority Health and Health Disparities entitled “*Training* ***LEAD****ers to Accelerate Global Mental Health Disparities Research”* (LEAD; T37MD014218) as an example of research training programs that have promise in effectively strengthening the pipeline of underrepresented minority scientists to advance research in global mental health disparities. The training program has the following specific aims:

**Aim 1:** To provide a research training program to five cohorts of advanced MD and PhD students, postdoctoral fellows, and junior faculty that equips trainees with foundational global research skills and knowledge through experiential learning, mentoring, “hands-on” immersion in domestic and international mental health studies, individualized consultation, feedback, goal setting and monitoring and web-based support across time.
**Aim 2:** To recruit five cohorts of advanced MD and PhD students, postdoctoral fellows, and junior faculty from underrepresented groups in the U.S. committed to conducting health disparities research, including a specific focus on global mental health prevention, intervention, services and implementation research within resource-constrained settings.
**Aim 3:** To evaluate the short-term and longitudinal outcomes of LEAD training program.

## Description of the Lead Global Training Program

LEAD is designed as a two-phase training program for advanced pre-doctoral students, post-doctoral trainees, and junior faculty from diverse backgrounds in the U.S., including groups underrepresented in biomedical, behavioral, clinical and social sciences research, interested in global mental health disparities research. All trainees are recruited from universities across the U.S. and are trained over an intensive 12-week program.

Phase 1 of LEAD consists of 4 weeks at Washington University in St. Louis for targeted skills and knowledge-building didactic seminars plus field-based research experiences within the St. Louis community. Phase 2 of LEAD consists of 8 weeks in a selected global site in a SSA country with research funded by the National Institutes of Health (NIH) that meets a trainee's research interest.

Each year, one to two postdoctoral LEAD trainees complete the 12-week training phases and transition into a 9-month fellowship at Washington University in St. Louis, with a potential for extension to a 2 year. The program uses a team based mentoring approach. Each trainee is matched with one U.S.-based mentor affiliated with LEAD and one SSA-based mentor, both of which will provide technical, research-focused support, but also ongoing career development guidance and support as well. Specifically, the proposed training program capitalizes on the rich resources for U.S. local and global health disparities (including mental health) available at Washington University in St. Louis, to provide trainees with the skills and experiences needed to lead multi- disciplinary, collaborative research teams focused on health disparities research in low-resource communities. The training program meets the non-human subjects research criterion.

### Phase 1. Didactic Training and U.S. Field-Based Research Mentorship

#### Phase 1a: Didactic Training

During Phase 1 of LEAD, all trainees participate in targeted skills and knowledge-building didactic training and seminars. Because trainees enter the program with different skills, strengths, and career levels, they are able to select from elective coursework that is currently being offered through summer programming at Washington University in St. Louis. Prior to the program, trainees work with their U.S. faculty mentors, to develop an Individual Development Plan to identify the most appropriate elective coursework for their career development. In addition to this tailored research experience, trainees are required to attend core seminars on various health disparity research topics, mental health seminars (including research topics focused on child and adolescent health and mental health) as well as career development workshops and seminars (e.g., grant writing, presenting research to a lay audience, etc.).

#### Phase 1b: Field-Based Research Mentorship

St. Louis, Missouri is an ideal domestic site for all LEAD trainees to increase knowledge in health disparities and social determinants of health *via* frontline fieldwork experiences. The St. Louis region is among the worst in the nation in regard to racial/ethnic and health disparities (14). The systemic issues impacting the St. Louis area are deeply rooted in inequities across educational opportunities, healthcare access, and economic opportunities that afflict the most vulnerable (15). Thus, trainees will be able to learn from faculty's ongoing research in St. Louis and apply lessons to other regions experiencing similar health disparities.

The primary focus of research training experience during Phase 1 is the apprenticeship model where the trainee's work will be focused on a single but broad ranging research project that works to strengthen the trainee's aptitude in health disparities research in pre-paration for their entry into Phase 2 of LEAD where the trainee will engage in field-based research, predominantly in the SSA region.

### Phase 2: Field-Based Research Experiences in SSA Region

Trainees will travel to SSA in Phase 2 (Weeks 5–12) to take advantage of the opportunities offered by three NIH-funded global research hubs across eight SSA countries. These three hubs are focused on scaling up mental health interventions in low-resource communities. Each of these hubs will provide opportunities for LEAD trainees to engage with and learn about global mental health disparities research, including but not limited to community engagement, instrument/intervention adaptation, intervention implementation, and data collection. It is important to note that all the SSA countries included as potential sites for the trainees are characterized by poverty, poor mental health services, a dearth of mental health researchers and practitioners, community violence, and civil unrest (16–21). Combined, these characteristics negatively impact overall communitie's mental health functioning and perpetuate a history of collective trauma over the life course and across generations. The studies associated with the sites are described in detail below:

**Strengthening Mental Health and Research Training in Sub-saharan Africa (SMART AFRICA) (U19MH110001, Ssewamala, McKay, & Hoagwood, MPIs):** SMART Africa Center is a transdisciplinary research center aimed at reducing gaps in child mental health service and research using a population approach. SMART Africa Center is one of the first child mental health implementation research partnerships located in SSA. The SMART Africa Center has a scale-up study in Uganda and pilot studies in Ghana and Kenya that examine the impact of an evidence-based multiple family group intervention that addresses serious child disruptive behavioral challenges as well as the multi-level (State/government, NGOs, families, schools, communities) influences on the uptake, implementation, effectiveness, and sustainability of evidence-based practices.

**Youth FORWARD: Capacity Building in Alternate Delivery Platforms and Implementation Models for Bringing Evidence-based Behavioral Interventions to Scale for Youth Facing Adversity in West Africa: (U19MH109989, Theresa S. Betancourt, PI):** Youth Functioning and Organizational Success for West African Regional Development *(Youth FORWARD)* is a mental health services research/ implementation science initiative involving local governments, non-governmental organizations, and academic institutions. Its dual focus is the science of scaling up evidence-based mental health care for high- risk youth in Sierra Leone and Liberia, and the expansion of local capacity to conduct and use further research. An implementation study takes place in Sierra Leone. Capacity-building activities occur in both countries. The scale-up study in Sierra Leone uses a hybrid implementation-effectiveness trial design across several youth entrepreneurship programs to evaluate an innovative approach to training and supervision and their influence on integration, fidelity, cost and sustainment of the Youth Readiness Intervention into a national youth employment program which is supported by major development actors in close coordination with local District Youth Councils.

**Southern African Research Consortium for Mental Health Integration (S-MhINT): Research and Capacity Building Consortium to Strengthen Mental Health Integration in South Africa, Mozambique and Tanzania (U19MH113191, Inge Petersen, Arvin Bhana, MPIs)**: S-MhINT is a research and capacity building consortium in Southern Africa that aims to strengthen regional mental health integration into primary health, antenatal, and chronic care platforms using implementation science in under-resourced areas of eastern South Africa, central Mozambique, and southern Tanzania. S-MhINT examines the multi-level influences on the uptake, implementation, effectiveness, and sustainability of an existing scale up of an integrated mental health package for chronic disorders at the primary health care level in two different districts having different resource capacities in South Africa. The project also aims at building implementation science and dissemination research capacity in the region, recruiting service providers, managers, and policy makers as trainees, providing real world opportunities, mentorship, and necessary knowledge to conduct optimal scale-up of evidence-based integrated mental health care.

### Adaptations to the LEAD Program to Mitigate the Impact of the COVID-19 Pandemic

Due to COVID-19, the LEAD curriculum shifted from a 4-week in-person training at Washington University in St. Louis to an online 10-week series of didactic training, program cohort meetings, and meetings with mentors. In addition, all travel was suspended. Hence, Phase 2 of the program was not implemented as described, but instead, trainees were able to work with their mentors to develop manuscripts out of existing data sets, work with mentors on COVID-related revisions to existing Institutional Review Boards, and attend community collaborative board meetings that were held virtually. In addition, we used this opportunity to expand research projects beyond the original proposed sites to include domestic mental health disparity studies.

The online format allowed us to expand our lecturing network to invite guest lecturers from across the U.S. and Uganda, resulting in a wider range of topics and perspectives. This format also enabled us to be responsive to feedback from the trainees in real time to incorporate additional research methodology content as needed for trainees and provided opportunities to discuss and develop curriculum around current event topics such as the COVID-19 pandemic.

Through the online platform, we brought the LEAD program together with our two other NIH-funded mental health-focused training programs (R25MH118935; D43TW011541) that ran concurrently. Bridging the three programs allowed our team to leverage additional resources, create additional opportunities for networking, bi-directional peer feedback and support from a larger cohort of trainees and faculty mentors. This additional cross-program collaboration resulted in one manuscript and two grant submissions involving trainees from different programs. Moreover, LEAD trainees had the opportunity to participate in an NIH mock review session as observers and peer reviewers for pilot grant applications from Researcher Resilience Training Program (R25MH118935) fellows, allowing them to gain valuable insight into the NIH review process and practice their own review skills as reviewers during the summer training period.

We anticipate using a hybrid didactic curriculum in the future, where we can add additional web-based programing, including useful skills-based seminars and supplemental group check-ins with all our trainees at various sites throughout Phase 2 of the program. These enhancements would not have been possible without the increased technological advancements at our program sites due to COVID-19 pandemic.

### Postdoctoral Research Training

In addition to participating in the 12-week program, the year-long postdoctoral training experience focuses primarily on research experiences in individual research laboratories, and the primary responsibility for the organization of the postdoctoral experience rests with the faculty mentor. Training of our year-long postdoctoral fellow(s) occurs *via* the following mechanisms: (1) Working with their mentor on an agreed upon research project in their Individual Development Plan; (2) Regular communication with faculty mentors at weekly meetings to conceptualize research questions, analyze datasets, and discuss results; (3) Establishing collaborative research projects with other research programs as well as networking with other postdocs at Washington University in St. Louis**; (**4) Regular career advice *via* mentorship meetings and more formal seminars; and (5) National networking opportunities through conference presentation opportunities.

### Trainee Recruitment and Selection Process

This program recruits 7–9 predoctoral and/or junior faculty and 1–2 postdoctoral trainees each year from underrepresented backgrounds in biomedical, behavioral, clinical and social sciences research, who are interested in health disparity research, with an emphasis on global health disparities.

#### Trainee Recruitment and Retention Plans

Washington University in St. Louis is diligent in our efforts to recruit outstanding underrepresented minority (URM) candidates. To select the most qualified applicants, we recruit underrepresented minority trainees focused on health disparity research from a range of local and national institutions. Efforts to recruit diverse candidates are aided by the increasingly diverse ethnic and cultural background of our training faculty and by the strong representation of highly productive researchers on our faculty.

We seek new recruitment sources for trainees through our large networks and at conferences. The Directors and TAC members lead targeted recruitment efforts within their own networks by contacting relevant colleagues, students, and student organizations. We also market to potential feeder programs by announcing the LEAD program in conferences, professional/academic associations, journals and newsletters with readers from under-represented populations.

Further, the LEAD program advertises widely among chairs of PhD and MD programs; Deans and Associate Deans of Research; Presidents of academic and professional organizations to be circulated in listservs; linkages with NIH staff; and other professional/academic networks.

#### Trainee Selection Process

The LEAD Program online application includes sections to describe the candidate, their career development plan, their preferred mentor(s), their research plan, Responsible Conduct of Research training and Human Subjects certification. In addition, applicants are required to provide two letters of reference as well as an NIH biosketch.

The review committee includes investigators from diverse disciplines who have experience mentoring trainees and reviewing applications. Any reviewer with an existing relationship with an applicant is recused during the discussion and scoring of that trainee. Similar to an actual NIH review, each application is reviewed by three reviewers. Reviewers summarize the strengths and limitations of the applicant and the proposal, score each section of the proposal, and give an overall score. Scores are calculated and applicants ranked accordingly. Following this scoring process, the top 12 candidates advance to the second round consisting of a phone interview with program directors and staff. These 15-min interviews allow for program leadership to access fit for the program and give trainees an opportunity to ask questions. In the case that the review committee cannot come to a consensus following these interviews, a secondary “council” review may be held by the Advisory Committee to determine funding based on overall scores and availability of resources.

#### Program Faculty Selection

**Figure 1:**
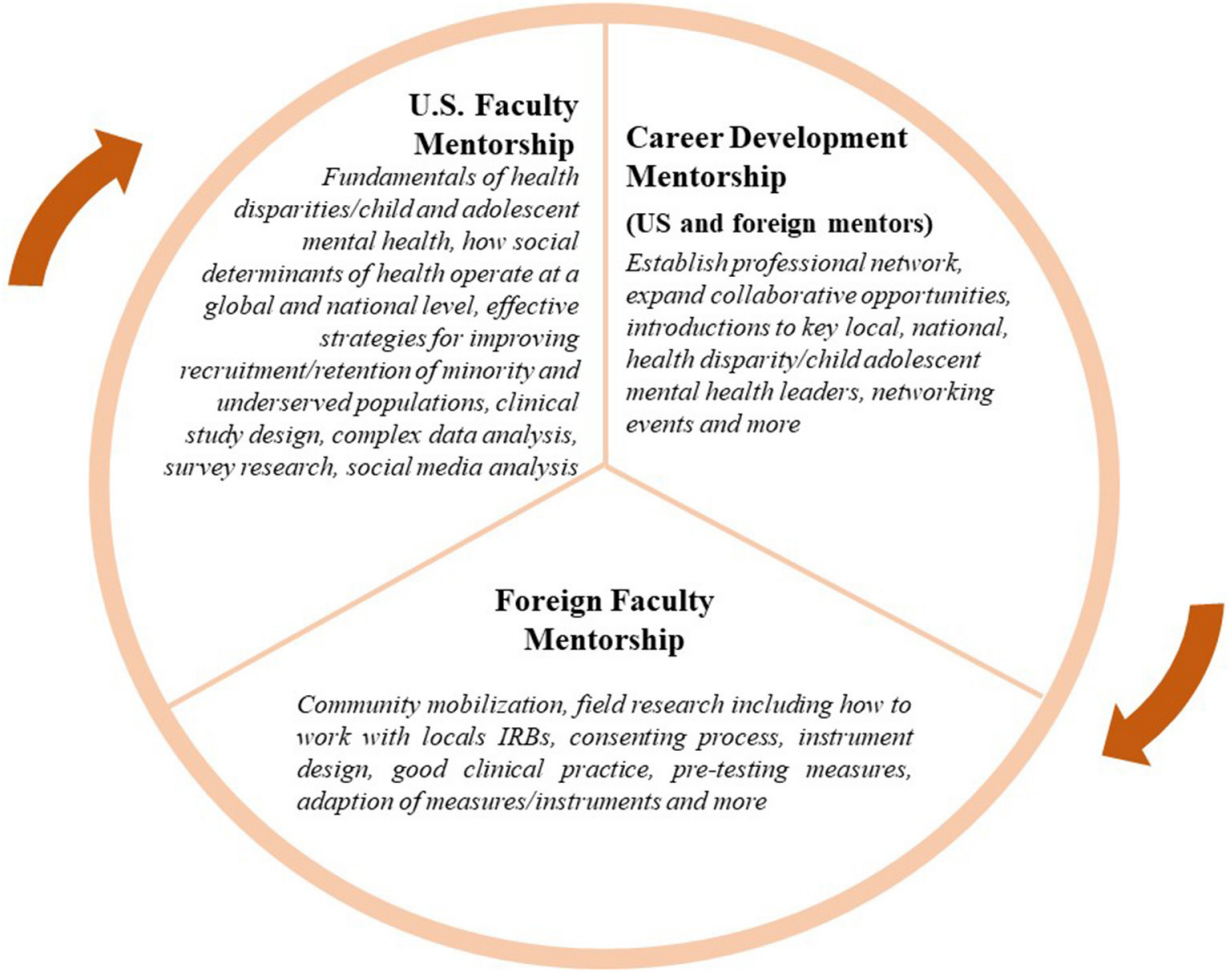
LEAD Mentorship Model.

Mentored research training is a critically important part of the LEAD Program. As such, we have assembled a group of established investigators with national and international recognition in global health disparities, mental health, public health, epidemiology, behavioral medicine, dissemination and implementation research, behavioral economics, and comparative effectiveness research. All LEAD program trainees are expected to have two mentors, a U.S. faculty mentor and a foreign faculty mentor (see Figure 1). Figure 1 highlights the unique and intersecting roles of the U.S. and foreign mentors.

Program mentors are expected to have a close working relationship with trainees, actively participate in guiding, reviewing and tailoring trainee's individual development plans and provide coaching, feedback, and direction to guide the career development and future success of LEAD trainees. To address varied learning styles and cultures of education, the mentoring model is informed by adult learning theories: Self-directed learning (22, 23), and Transformative learning (24, 25) theories. In addition, mentoring activities are informed by anti-oppressive theory (26–28), to create a culturally-sensitive, contextually-relevant, and inclusive learning environment. This is achieved by ensuring a learning environment that balances different learning styles and cultures through: (1) collaborating to select methods, materials, and resources; (2) developing learning objectives based on each learner's needs, interests, and skill levels; (3) designing collaborative and individual tasks; 4) encouraging reflective and discussion activities; (5) using visual, written, experiential, and other types of learning styles; and (6) evaluating and adjusting the quality of the learning experience while assessing further learning needs.

#### LEAD Mentor Training

Both U.S. and foreign faculty mentors are required to participate in a half-day web-based seminar led by the Program Directors. Key topics such as establishing expectations, effective communication, assessing understanding and fostering independence are covered and reference materials provided. In addition, mentors must sign “Mentor Agreements” describing expectations, time commitment, and requirements to participate in program development and evaluation. Mentors will be formally evaluated mid-way through the training period and following each summer session by their trainee(s) and reviewed by the Training Advisory Committee (TAC). If a problem with a mentor occurs, the LEAD Directors will intervene to facilitate the process of decision making and solutions, provide support, and arrange for new or additional mentors if necessary. Mentors with negative evaluations or whose circumstances change such that they no longer fulfill the criteria to be a mentor will be removed from the pool.

#### Administrative Structure

LEAD is led by a multidisciplinary team of accomplished investigators. Leadership is supported by 10 TAC members comprised of accomplished leaders in the field of global mental health and mental health disparities research, including practitioners, policy makers, and/or **biomedical, behavioral, clinical and social sciences researchers** with extensive experience running training programs and/or experience in mentorship and career development (see https://sites.wustl.edu/lead/ for more details). Members of TAC are expected to serve for 5 years. They provide strategic guidance and oversight for activities of the Leadership team including candidate selection, scientific review of trainee projects, evaluation of trainees, training program progress, program development, implementation and evaluation.

The TAC meets annually *via* web-conferencing to plan and develop the program content, curriculum, and competencies. In addition to providing overall strategic direction to the program, they give seminars to our trainees, assist with program marketing and candidate selection, and review our program and outcomes. Program recommendations are received both verbally and *via* written correspondence.

## Training Program Evaluation

To assess program effectiveness, scholars, mentors and program curricula are regularly evaluated and the results of these evaluations are used to improve our LEAD training program. The evaluation is an ongoing process and the overall impact of the LEAD program will be published once data has been collected from all cohorts recruited during the 5-year period. The program and curricula are evaluated using the following four metrics: (1) Tracking the number and types of scholars applying, accepted, matriculating, and completing the training; (2) Measuring scholar satisfaction with the training programs *via* course evaluations and mentor evaluations; (3) Scholar outcomes, and scholar productivity (publications, grants, academic appointments, and promotions); and (4) Regular meetings with the TAC to critically review programs, curricula, and practices. Feedback is given in the form of suggestions for improvement. A variety of processes and outcome measures are used to determine the effectiveness of curricula and mentored research experiences. Evaluation materials include the following:

### Course Evaluations

Courses are evaluated *via* direct observation by Program Directors, evaluations of final projects, and written evaluations by both trainees and their mentors. The evaluation questions assess clarity of expectations, organization and content of lectures and materials, topic relevance, instructor engagement, encouragement of critical thinking, and global assessment of the instructor. Coursework and program data also are collected from Qualtrics surveys sent out immediately following each training session and trainee exit interviews. The Qualtrics surveys consist of both quantitative and qualitative aspects and included a 5-point Likert Scale evaluating the sessions “Relevance of the topic to my research;” “Overall quality of the speaker(s) and their presentation(s);” and the “Convenience of event time/schedule.” In terms of qualitative questions, trainees were asked what they “liked and found useful” and “how the session could be improved.”

The exit interviews are 30-min sessions with an independent coordinator that query program strengths and weaknesses *via* 10 open-ended questions. Examples include “How helpful were the training topics to you in the research setting? Were there aspects of research and research careers that you wish had been covered in the training webinars?” and “Please discuss your mentored research experience. What were the positives and negatives of your experience?” Surveys and exit interviews are used to evaluate quality, accessibility, coherence, additional research topics that should be added to the program, and access to research support.

### Mentor Evaluations

At the 5-week point and end of the summer program trainees complete online Qualtrics surveys to evaluate their relationship with their mentor. In addition to questions asking about the number of meetings and communication trainees have with their mentors each week, the evaluation includes a 7-point Likert Scale in order to gauge each mentor's time commitment, availability, and effectiveness as well as determine specific problems and strengths of the relationship. Questions include the following: “My mentor is supportive and encouraging;” “My mentor provides constructive and useful critiques of my work;” “My mentor is helpful in providing direction and guidance on professional issues (e.g., networking);” “I have access to appropriate resources (e.g., experts, electronic contacts, source materials);” and “I feel challenged to extend my abilities (e.g., risk taking, try a new professional activity, draft a section of an article).” There are also five open ended questions designed to summarize overall progress and potential barriers including: “List any barriers to meeting your projects goals” and “How can your mentor, the directors, and/or LEAD program staff help support your work?” If problems arise, the Program Director will schedule meetings with both parties and if necessary, propose a new mentor.

### Trainee Evaluations

Mentors will evaluate trainees both during the mid-point and at the end of the summer program using online surveys in order to identify possible problems and redirect trainee efforts when necessary. Similar to the mentor evaluations listed above, this approach uses a 7-point Likert Scale with the following questions: “Incorporates constructive and useful critiques into their work;” “Appropriately asks for direction and guidance on professional issues (e.g., networking, politics);” “Responds to my questions satisfactorily (e.g., timely response, clear, comprehensive);” “Has been productive in their research;” and “Is on track to meet their IDP goals.” Mentor feedback also includes open ended questions to summarize overall progress and areas of concern. This feedback is shared with the TAC and with the trainees to help guide career development.

### Trainee Tracking

Trainees are tracked for 15 years after completing their program to obtain long-term feedback and insights on program benefits, pitfalls and effectiveness. The program coordinators monitor scholar employment, career development, and milestones, including research positions, faculty appointments, grants awarded, presentations, publications, awards, participation in study sections, and mentoring record.

### Bibliometric Analysis

Working with the CPHSS, and the Becker Library, LEAD Program Directors will use social network analysis to assess scholarly collaboration to research productivity.

The results of these evaluation tools are entered into a database to generate reports and track long-term outcomes.

**Figure 2:**
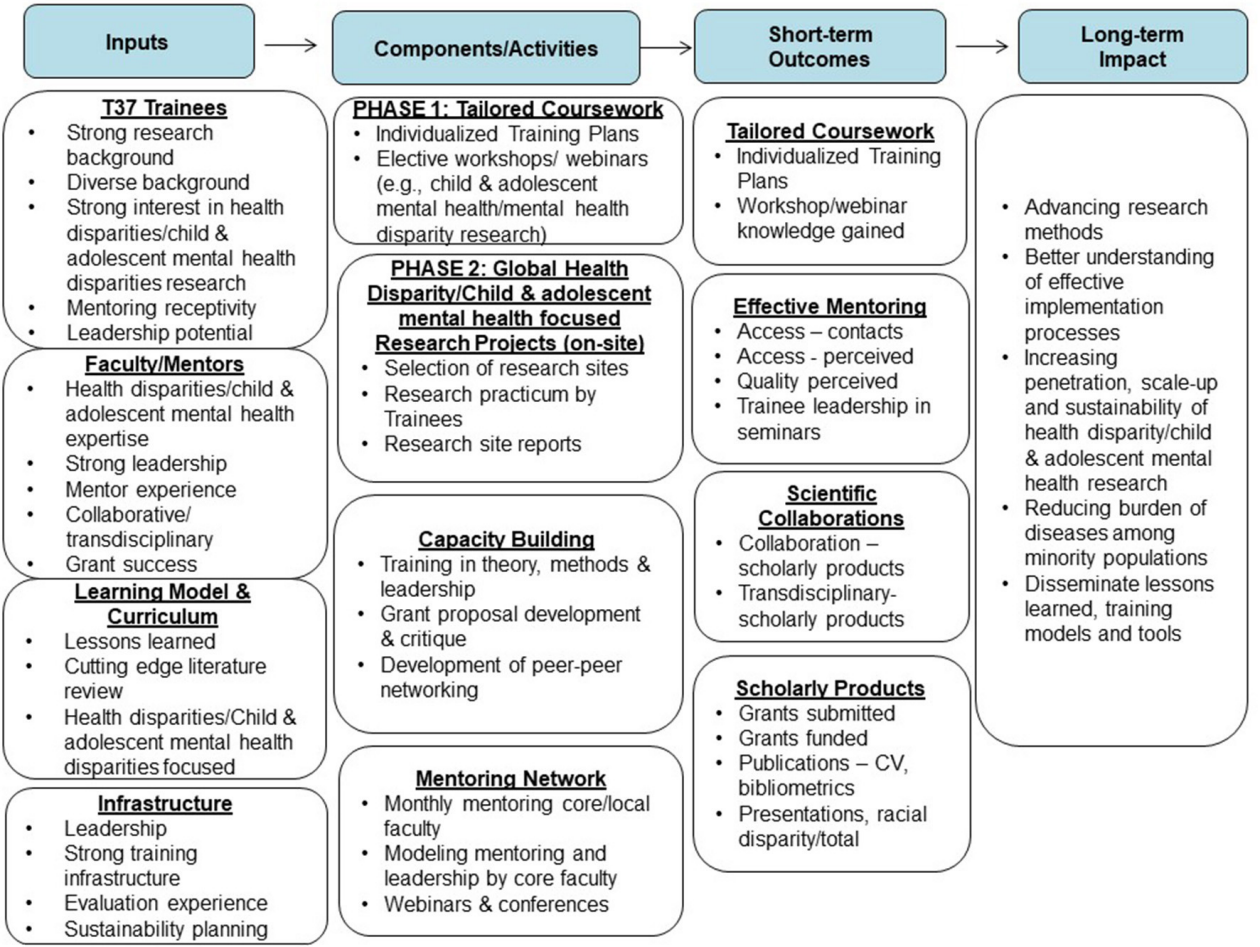
LEAD Program Logic Model.

As shown in the Program Logic Model (see Figure 2), program outcomes are measured by the number of funded research grants, presentations, and publications for scholars, faculty, and mentors, and the relevance of these products for the specific research area. Additional variables can be added, although the basic evaluation materials and core variables will be maintained so that tracking and evaluation results for all programs can be pooled, which improves the overall long-term evaluation of all programs.

### Preliminary Findings

Our preliminary findings suggest that the training program has been successful and is well received by the trainees. For instance, between June 2020—September 2021, our 19 LEAD trainees have published 19 peer-reviewed manuscripts, presented at 19 academic conferences, and received two grants, including an NIH-funded R01 study. Results from our two cohorts indicate that overall, the training curriculum received very high ratings, with 83% of Summer 2020 trainees and 91% of Summer 2021 trainees ranking the “Overall quality of the speakers and their presentations” as excellent or very good. One noted, “I am very interested in qualitative research and found this very informative…. [I appreciated hearing] both a Ugandan and American perspective. Both presenters were very knowledgeable and experiences and gave examples from their research experience.” Both cohort evaluations also indicated a high level of communication between mentors and trainees, with meetings occurring at least once per week and multiple electronic communications reported by all respondents. In general, trainees indicated they had the mentor support and materials they needed to accomplish their work.

## Conclusion

The overall goal of the LEAD Global Training Program is 2-fold: (1) increase the number of underrepresented minority researchers from the U.S. trained in conducting mental health disparity research; and (2) create a cadre of rigorously trained researchers who will focus on global and U.S. mental health disparities, especially in resource-constrained settings, where mental health disparities are understudied.

LEAD seeks to provide a robust platform for the development and implementation of evidence-based interventions in global mental health disparities–led by culturally-conscious investigators from underrepresented minority groups. While federal funding agencies, such as NIH, are committed to diversifying the workforce and increasing the proportion of federally-funded investigators from underrepresented populations, racial and ethnic diversity of applicants remain limited. For instance, <5% (~4.7%) of the NIH applications come from Black (1.4%), Hispanic (3.2%), and Native American (0.05%) investigators. Compared with NIH R01 applications from white investigators, applications from black investigators are 13.2 percentage points less likely to be awarded. Recent reports indicate no major improvements in these numbers, where African American/Black scientists make up only about 2% of the R01 application pool in 2020 (29) and underrepresented minority investigators only make up 5.9% of the R01 awardees. Hence, it is critical to invest in researchers from underrepresented backgrounds.

There has been a call for shared lessons and efforts, arguing that lessons learned from global mental health efforts in low-income countries dominated by the global south, including SSA, may be relevant to addressing global mental health disparities, and improving overall care for vulnerable populations in high-income countries, including the U.S. (13).

The LEAD program is purposefully designed to disrupt the traditional practice of researchers from the Global North “teaching” researchers from the Global South. The program recognizes and values the wealth of experience and expertise of the SSA researchers in addition to the importance and applicability of lessons learned from the region in low-resource settings in the U.S. In addition to Global South researchers leading many of the didactic training sessions and panel discussions during the summer programming, LEAD matches trainees with senior investigators from the Global South to immerse them in intensive field-based research on the continent.

In short, the LEAD program aims to ultimately provide a robust platform for the development and implementation of evidence-based interventions in global mental health disparities–led by culturally conscious investigators from underrepresented minority groups.

## Author Contributions

PC-R and FS are the principal investigators of the training program. OSB is a co-investigator on the training program, contributed to the conceptualization of the training program and drafted this manuscript. BA and LP are the training managers for the program and contributed to the drafting of this manuscript. PN is a co-investigator and contributed to the drafting of this manuscript. LS, TB, AB, and TE are members of the Training Advisory Committee and contributed to the drafting of this manuscript. All authors reviewed and approved the submitted manuscript.

## Funding

The LEAD training program was funded by the National Institute on Minority Health and Health Disparities (T37MD014218; MPIs: Ssewamala, Cavazos-Rehg). The training program was partially supported by the National Center For Advancing Translational Sciences (UL1TR002345; PI: Powderly). The content is solely the responsibility of the authors and does not necessarily represent the official views of the National Institutes of Health.

## Conflict of Interest

The authors declare that the research was conducted in the absence of any commercial or financial relationships that could be construed as a potential conflict of interest.

## Publisher's Note

All claims expressed in this article are solely those of the authors and do not necessarily represent those of their affiliated organizations, or those of the publisher, the editors and the reviewers. Any product that may be evaluated in this article, or claim that may be made by its manufacturer, is not guaranteed or endorsed by the publisher.
